# Visualizing arthritic inflammation and therapeutic response by fluorine-19 magnetic resonance imaging (^19^F MRI)

**DOI:** 10.1186/1476-9255-9-24

**Published:** 2012-06-21

**Authors:** Anthony Balducci, Brooke M Helfer, Eric T Ahrens, Charles F O’Hanlon, Amy K Wesa

**Affiliations:** 1 Department of Research and Development, Celsense, Inc., Pittsburgh, PA 15222, USA; 2 Department of Biology, and Pittsburgh NMR Center for Biomedical Research, Carnegie Mellon University, Pittsburgh, PA 15213, USA

**Keywords:** Inflammation, Monocytes, Macrophages, Magnetic resonance imaging (MRI), Biofunctional imaging, Perfluorocarbon, Contrast agent, Arthritis

## Abstract

**Background:**

Non-invasive imaging of inflammation to measure the progression of autoimmune diseases, such as rheumatoid arthritis (RA), and to monitor responses to therapy is critically needed. V-Sense, a perfluorocarbon (PFC) contrast agent that preferentially labels inflammatory cells, which are then recruited out of systemic circulation to sites of inflammation, enables detection by ^19^F MRI. With no ^19^F background in the host, detection is highly-specific and can act as a proxy biomarker of the degree of inflammation present.

**Methods:**

Collagen-induced arthritis in rats, a model with many similarities to human RA, was used to study the ability of the PFC contrast agent to reveal the accumulation of inflammation over time using ^19^F MRI. Disease progression in the rat hind limbs was monitored by caliper measurements and ^19^F MRI on days 15, 22 and 29, including the height of clinically symptomatic disease. Naïve rats served as controls. The capacity of the PFC contrast agent and ^19^F MRI to assess the effectiveness of therapy was studied in a cohort of rats administered oral prednisolone on days 14 to 28.

**Results:**

Quantification of ^19^F signal measured by MRI in affected limbs was linearly correlated with disease severity. In animals with progressive disease, increases in ^19^F signal reflected the ongoing recruitment of inflammatory cells to the site, while no increase in ^19^F signal was observed in animals receiving treatment which resulted in clinical resolution of disease.

**Conclusion:**

These results indicate that ^19^F MRI may be used to quantitatively and qualitatively evaluate longitudinal responses to a therapeutic regimen, while additionally revealing the recruitment of monocytic cells involved in the inflammatory process to the anatomical site. This study may support the use of ^19^F MRI to clinically quantify and monitor the severity of inflammation, and to assess the effectiveness of treatments in RA and other diseases with an inflammatory component.

## Background

Rheumatoid arthritis is a systematic, chronic, debilitating disease which affects approximately 0.5-1% of the world population [1,2]. Inflammation of the synovial membrane is a hallmark of the disease, with the disease eventually progressing to cartilage and osseous degradation. There is no known cure, however therapeutic treatments are available and recent advances in the imaging of the disease and associated inflammation have allowed earlier diagnosis and intervention [3,4], with the possibility for increased mobility and quality of life for patients through disease management [5-7].

Imaging for arthritis, or inflammation in general, can be classified as either anatomical imaging, where the manifestations of the disease on the body are observed, or biofunctional imaging, where the biological processes involved in the disease are observed [8]. In the case of RA, imaging for clinical diagnosis is limited to anatomical imaging of bone erosion (MRI, CT), inflammation of the synovial membrane (ultrasound, MRI), and joint effusion and tissue swelling (x-ray) [9,10]. These techniques, though useful, often only elucidate the disease after permanent damage is done, limiting the applicability of early intervention therapies [11]. Biofunctional imaging of arthritis focuses on metabolic activity, cellular infiltrates, and cytokine production [10], which often occur prior to the onset of permanent anatomical damage due to the disease. It may serve as an indicator of the presence of disease and severity, enabling earlier diagnosis and treatment [3]. By example, in RA patients with clinically stable disease, synovitis may persist, leading to disease progression [12]. Methodologies for non-invasive detection and localization of inflammation in RA include PET/CT [13], ultrasound [14], optical (fluorescence) imaging [15], and MRI.

MRI is of particular interest due to its high spatial resolution, which allows precise anatomical visualization of bone degradation, and its current role in the diagnosis of RA [16]. Furthermore, the safety profile of MRI makes it amenable to repetitive imaging sessions, an important consideration for use in a prolonged, chronic disorder. MRI images of macrophage infiltration associated with inflammation have been obtained in a variety of disease states using transition metal and super paramagnetic iron oxide (SPIO) contrast agents [17]. Gadolinium has been used as a blood-pool marker of sites of inflammation [18,19] and quantitative methods using the reagent have been developed [20-22]. SPIO nanoparticles are phagocytosed by circulating monocytes/macrophage, and provide MR contrast when those cells aggregate at the site of inflammation [23-27]. Unfortunately, metal-based MR contrast agents operate by either increasing or decreasing the signal obtained in the MRI image, effectively convoluting the anatomical image with cell-level information and impeding normal observation of disease progression with the technique. The use of an alternate nucleus, such as fluorine MRI (^19^F MRI) avoids this difficulty by specific detection of the fluorine atom, providing a signal which varies in a direct relationship with the amount ^19^F present, without any background signal from host tissue, and without distorting the anatomical ^1^H image.

^19^F MRI with the use of a perfluorocarbon (PFC) contrast agent has emerged as a powerful technique through the in situ labeling of circulating macrophage and monocytes. Labeled inflammatory cells traffic to sites of inflammation where they accumulate and render those tissues detectable by ^19^F MRI. This approach has been used as an indicator of inflammation in a variety of disease models including experimental autoimmune encephalitis [28], allograft rejection [29,30], inflammatory bowel disease [31], abscess visualization [32], pulmonary inflammation [33], and post-ischemia inflammation in the heart and brain [34]. Un-inflamed tissues (with the exception of the reticuloendothelial system) lack ^19^F signal, and therapeutic intervention can modulate the ^19^F intensity [31,33], indicating the specificity of this approach for inflamed tissues. Furthermore, the ^19^F signal correlates with the degree of macrophages present in the inflammatory site [28]. However, no correlation to disease severity has yet been established through co-measurement of ^19^F MRI and with a clinical marker.

Here, we employ a well-known model of RA, with a facile, independent measurement of clinical disease progression (ankle diameter), to validate the ability of ^19^F MRI to ascertain disease presence and severity. The current study has two aims: (1) to evaluate the ability of ^19^F MRI to quantitatively measure disease severity relative to standard measurements, and (2) to determine whether serial imaging with ^19^F MRI reflects the course of disease progression or response to therapy.

## Methods

### *Animals, arthritis model and treatment*

**Figure 1:**
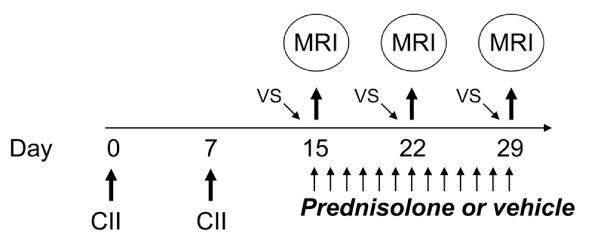
**Study schema for serial monitoring of arthritic inflammation & disease.** Six rats were injected intradermally with Type II collagen (CII) in incomplete Freund’s adjuvant on days 0 and 7. Animals were randomized into study cohorts (3 per group) on day 13, and all injected with PFC contrast agent. Two days later animals were imaged by ^1^H ^19^F MRI, and placed on daily vehicle or prednisolone for the duration of the study. MRI was repeated at weekly intervals 48 hours after contrast agent administration. Clinical measurements of ankle swelling and animal weight were conducted from day 15 (after the initial collagen immunization) to study end, every 3 to 4 days.

All animal studies were conducted with Institutional IACUC approval at a contract research organization (Covance Laboratories, Greenfield, IN). Female Lewis rats were purchased from Charles River, and housed under specific pathogen-free (SPF) conditions. The rat collagen induced arthritis (CIA) model was conducted as previously described [35,36]. Briefly, Lewis rats received two weekly doses of Type II bovine collagen (CII, 2 mg/mL) in incomplete Freund’s adjuvant, intradermally, leading to the onset of arthritis with clinical symptoms appearing by day 14 after the initial immunization, peak swelling in the limb joints by day 21 and arthritic damage by day 28. Naïve animals served as controls, and were housed until day 28. The development of arthritis and severity was measured by paw swelling in the hind limbs using caliper measurements every 3-4 days. To determine whether ^19^F MRI could be used to monitor the effect of drug therapy on established inflammation, CIA animals were randomized upon disease onset (day 14) into cohorts of vehicle control or drug treatment for monitoring by serial imaging. Therapy consisted of daily prednisolone (Sigma Chemical Company, St. Louis, MO; 10 mg/kg, selected as a model therapeutic due to its efficacy in treating existing disease) or vehicle control (1% hydroxyethylcellulose, 0.25% tween-80, 0.05% antifoam), dosed daily by oral gavage (3.4 ml/kg). The PFC contrast agent was administered prior to each imaging session to evaluate the active inflammatory response at the time of imaging. A schema of the study design is shown in Figure 1. 

### *Magnetic Resonance Imaging*

For in vivo imaging studies V-Sense, a sterile PFC-containing emulsion (20% (v/v) of perfluoropolyether, VS-1000H, Celsense, Inc., Pittsburgh, PA) was used as a ^19^F contrast agent to detect macrophage activity [37]. Forty-eight hours prior to imaging, a single 1.5 mL dose was administered intravenously through the tail vein.

MRI was conducted using a Varian 7T DirectDrive MRI spectrometer (Agilent Technologies, Santa Clara, CA) equipped with VnmrJ 2.2 C acquisition software, RHEL 4.u.3 OS, the Magnex 205/120/HD gradient set, a 35 mm i.d. transmit/receive volume coil, tunable for ^1^H or ^19^F imaging (m2m Imaging Corp., Cleveland, OH) and a physiological monitoring system (Small Animal Instruments, Inc. Stony Brook, NY). Rats were anesthetized with 5% isoflurane and maintained with an anesthesia nose cone at 1.5% isoflurane in oxygen. Prone rats were positioned with hind limbs extended and an external reference tube (containing 1:15 dilution of the PFC contrast agent in 1% agarose gel, and a known number of ^19^F atoms (i.e., spins/mm^3^) to enable quantitative measurement of fluorine content) placed between the legs, affixed to the animal cradle, and guided into the RF coil. Respiration and body temperature were monitored throughout image acquisition, and bore temperature was maintained below 30C.

The ^1^H image was obtained with a fast spin-echo sequence, multislice (21 slices, 1 mm thick), and high-resolution axial images along the length of the hind limbs, rostral and caudal to the ankles. The acquisition parameters were: repetition time/echo time (TR/TE) = 2000/22 ms, using a rapid acquisition with refocused echo (RARE) sequence, RARE factor = 8, 256 × 256 matrix, field of view (FOV) = 40 x 40 mm^2^, 2 averages, total acquisition time 2.1 minutes. For ^19^F images, a RARE sequence was used with TR/TE = 1800/10.1 ms, RARE factor = 8, 128 × 64 matrix zerofilled to 256 x 256, FOV 40 x 40 mm^2^, 128 averages, 21 slices and a total acquisition time of 30.7 minutes. The Larmor frequencies of ^1^H and ^19^F differ by ~6%.

### ^*19*^*F MRI data analysis*

Each MR imaging session included a reference tube containing a known dilution of the PFC contrast agent prepared at a concentration of 2.76 x 10^17^ spins/mm^3^. Voxel Tracker™ software (Celsense, Inc.) was used to correct for the effects of the Rician noise distribution inherent in MRI and to compare signal intensity from a region of interest to that of the reference tube to allow determination of the total amount of fluorine [38-40]. Regions of interest (ROIs) were drawn in the (1) noise region (2) reference material regions (3) right/left leg in each image slice. In most instances, no signal was observed in the first or last slice, indicating that the majority of the signal was within the area of analysis.

### *Histology*

On day 29, after the final scan, animals were anesthetized by CO_2_ inhalation and euthanized by cervical dislocation, and ankles and knees fixed in 10% neutral buffered formalin, then decalcified prior to embedding in paraffin. Sections were stained with hematoxylin/eosin to assess inflammation/cellular infiltration within the joints. Images were captured with an Olympus Provis light microscope, and sections were also digitized with a microscope slide scanner.

### *Statistical analysis*

Dunnetts’ method was used for comparisons of multiple cohorts. Two factor ANOVA was used to evaluate significance of longitudinal and treatment differences, followed by ad-hoc comparison of means using Tukey’s method. Paired T-tests were used to evaluate differences in the same animal at different time points, and unpaired T-tests were used to evaluate differences between cohorts. Error bars represent standard deviation.

## Results

**Figure 2:**
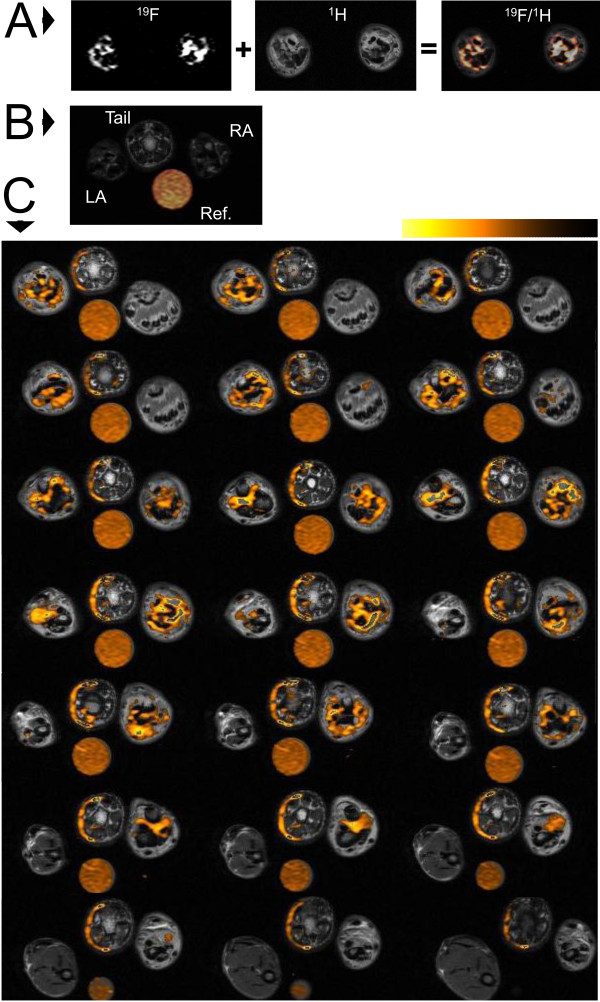
**Representative**^** 1**^**H and **^**19**^**F MRI overlays of rat ankles.A.**^19^F signal is rendered in hot orange scale and overlayed on a grayscale ^1^H image in this representative slice from an arthritic rat. **B.** Representative slice from a naïve rat indicating the placement of right ankle (**RA**) left ankle (**LA**), tail and reference tube (**Ref**). **C.** Complete series of ^19^F slices obtained through the ankles of a representative arthritic rat on day 15.

To determine whether ^19^F MRI could be used to image inflammation in arthritis, a cohort of rats were immunized twice with type II collagen to induce disease, and then dosed with the PFC contrast agent two days prior to ^19^F MRI. The time point for imaging (day 15) was selected as it coincided with nearly complete disease onset (90%) as measured by ankle swelling. Following MRI acquisition, ^19^F images were rendered in hot-orange pseudocolor and overlayed on the anatomical ^1^H image using Voxel Tracker™ software. A representative overlay is shown in Figure 2A. While all CIA rats had a strong ^19^F signal surrounding the bones in the ankles on day 15, consistent with the presence of inflammation at those sites, naïve rats did not have any detectable ^19^F in the hind limbs (Figure 2B), consistent with an absence of inflammation. Figure 2C depicts the entire panel of overlayed 1 mm axial slices through the hind limbs obtained from a single representative diseased rat.

**Figure 3:**
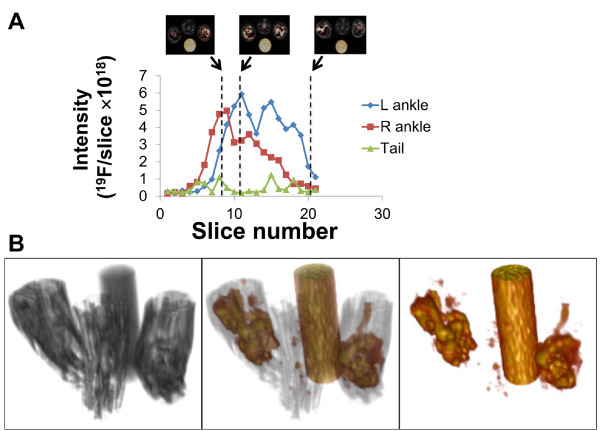
**Quantification and 3D rendering of inflammation obtained from**^** 19**^**F signal in rat ankles.A.** ROIs were drawn around each ankle and the tail in an image stack from a representative scan of an arthritic rat (day 15) to enable the calculation of ^19^F spins per slice. The reference tube containing a known concentration of ^19^F was used in calculations. Results are represented as the amount of ^19^F per slice in each of the ankles or tail, with images depicted for slices 7, 9 and 17. **B.** 3D rendering of spatial accumulation of ^19^F signal in the ankles of a representative arthritic animal (day 15). ^1^H image is depicted on the left, and fused ^19^F/^1^H image in the middle, and ^19^F on the right.

A comparison of ^19^F signal emanating from a region of interest with the known concentration of fluorine in the reference enabled the calculation of ^19^F nuclei per slice (Figure 3A) to identify the major sites of inflammatory infiltrates which have taken up ^19^F along the axis of the ankle. A 3D reconstruction of the distribution of ^19^F signal is depicted in Figure 3B.

**Figure 4:**
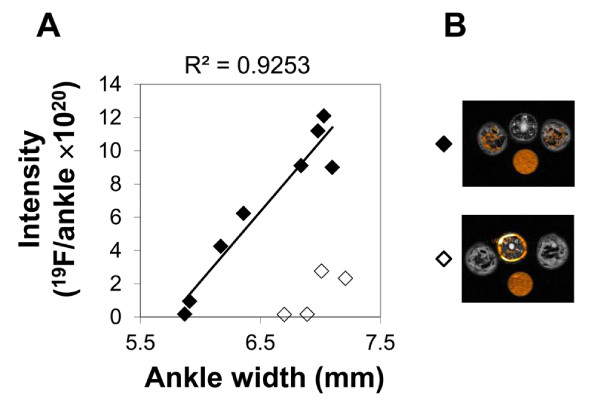
**Correlation between clinical measure of disease and**^** 19**^**F MRI imaging.A.** Clinical measurements of disease severity manifested as ankle swelling were obtained through caliper measurements of ankle thickness and were plotted versus the total ^19^F spins/ankle as measured from the MRI images on day 15 (◆). Solid line represents the linear trend generated from data on individual ankles (n = 12). Two animals had very high levels of ^19^F found in the tail near the site of tail vein injection, suggesting that V-Sense was not delivered into the circulation. These two animals (◇) had significantly lower levels of ^19^F in each ankle than animals with similar clinical measurements (p < 0.001), and were not included in the linear regression. **B.** Representative slices from animals without (◆) and with (◇) high ^19^F in the tail are shown.

In order to correlate ^19^F signal with the clinical measurement, caliper measurements were obtained from diseased animals at the time of imaging. Animals imaged prior to disease onset on day 10 exhibited no swelling in the hind limbs and also had no detectable ^19^F signal (n = 3, data not shown). In animals with clinically evident disease imaged on day 15, four of six animals exhibited a strong linear dependence between diameter and ^19^F intensity (R^2^ = 0.93), while two of the six animals exhibited swelling with a lower, but still detectable, content of ^19^F. In these two animals, a very strong ^19^F signal was observed in the tail near the injection site (Figure 4B), in contrast with the other four animals, where little ^19^F was detectable in the tail. A strong correlation (*R* = 0.96) was observed between the caliper measurement of the ankle and the total amount of ^19^F signal (Figure 4A) when the animals with high signal in the tail were excluded from analysis.

**Figure 5:**
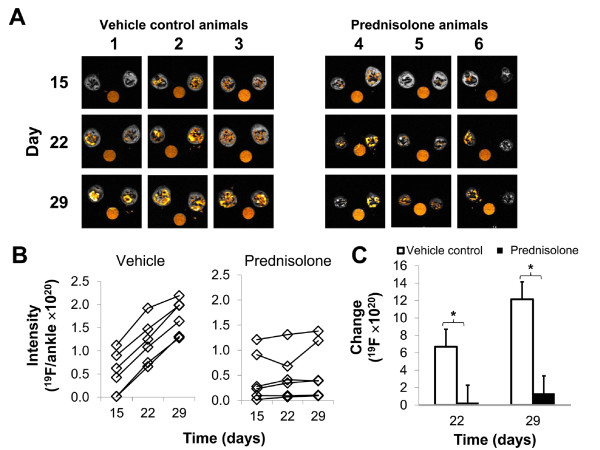
**Serial imaging and quantification of inflammation and therapeutic response in arthritic rats.** As depicted in Figure 4, arthritic rats undergoing steroid treatment or vehicle control underwent serial imaging with PFC contrast agent and ^19^F MRI. **A.** Representative slices from each of three control animals (1, 2, 3) and each of three treated animals (4, 5, 6) are depicted on days 15, 22 and 29. Each column represents slices from a single, serially imaged animal over time. **B.** The degree of inflammation as assessed by quantification of the concentration of ^19^F per limb was depicted over time in vehicle (left) and prednisolone treated (right) animals. **C.** The average change in ^19^F signal from t_i_ (day 15, the initial imaging session) to t_n_ (day 22 or day 29) in untreated (open columns) and prednisolone treated (filled columns) at days 22 and day 29 are shown. *Statistically significant as determined by unpaired *T*-test (*P* =7.29 x10^-6^ at day 22 and *P* =1.65 x10^-8^ at day 29).

**Figure 6:**
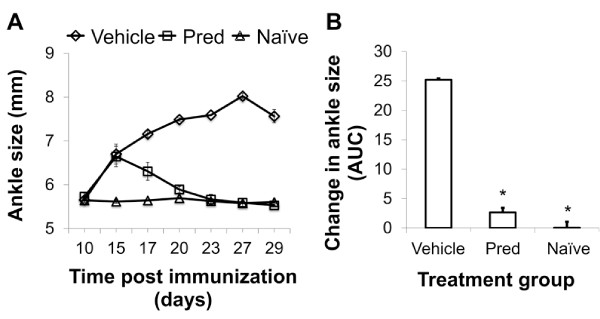
**Clinical measurements of ankle size.A.** Ankle swelling in 3 cohorts, naïve (Δ), diseased treated (□) and diseased vehicle control (◇) are plotted over time. **B.** Change in ankles size expressed as area under the curve (AUC). * *P* < 0.0001 as compared to vehicle control using Dunnett’s method.

**Figure 7:**
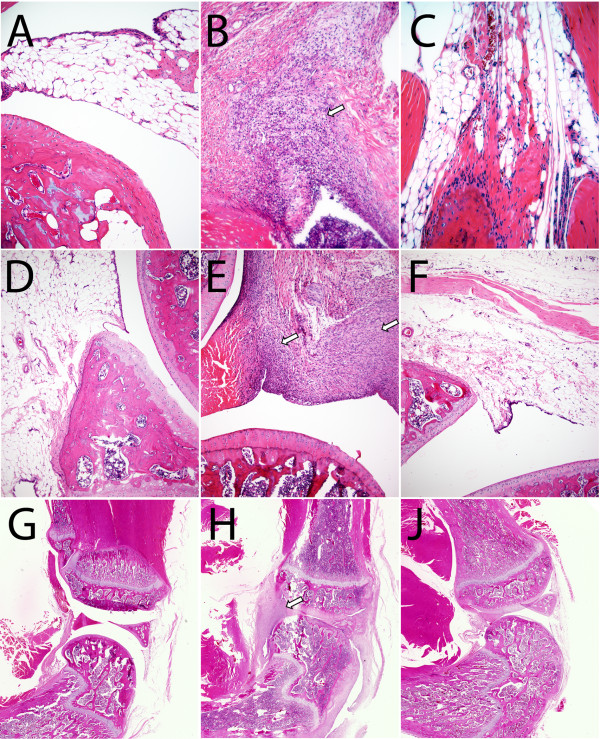
**Representative hematoxylin- and eosin-stained histologic sections of rat knees and ankles on day 29.** Each cohort was evaluated and examples are depicted of naïve controls (**A**, **D**, and **G**), vehicle-control treated CIA rats (**B**, **E**, and **H**) and prednisolone-treated CIA rats (**C**, **F**, and **J**). Images include photomicrographs of ankles (**A**, **B**, and **C**; 20X objective) and knees (**D**, **E**, and **F**; 10X objective) and knees taken with a slide scanner (**G**, **H**, and **J**). Inflammatory cell infiltration (arrows) is found in vehicle-control treated CIA rats.

To determine whether ^19^F MRI could be used to evaluate the course of disease (progression, or remission), cohorts of animals were serially imaged at weekly intervals following the onset of disease. At day 15, all animals had ^19^F signal in one or both of their ankles, as shown in Figures 5A (average signal of all ankles 4.87 x 10^19^ ± 4.50 x 10^19^ range: 1.66 x 10^18^ to 1.12 x 10^20^). Evaluation of the ^19^F signal over time indicated a significant difference among groups (Additional file 1: Table S1, *P* = 3.47 x 10^-4^). Upon treatment with the steroid prednisolone, no significant increase in ^19^F signal was detected in the hind limbs at day 22 and only a minor increase over day 15 was observed by day 29, suggesting that the therapy was effective in eliminating the additional recruitment of inflammatory cells at later time points (Figure 5B, Additional file 1: Table S1). In strong contrast there were highly significant increases in ^19^F signal detected in the vehicle control cohort, consistent with progressive disease and the persisting recruitment of inflammatory phagocytic cells (Figure 5B, Additional file 1: Table S1). While the ^19^F intensity was variable among individual animals (Figure 5B), serial evaluation of the change in signal revealed that animals with untreated progressive disease accumulated significantly more ^19^F signal as compared to the prednisolone group (Figure 5C; *P* = 7.29 x 10^-6^ at day 22; *P* = 1.65x10^-8^ at day 29). Clinical evaluation of hind limb swelling corroborated the imaging observations of the effectiveness of prednisolone for reducing swelling versus the control group (Figure 6A, B). To validate the efficacy of treatment, animals were sacrificed on day 29, and ankles and knees were fixed and paraffin-embedded for histological assessments. As shown in Figure 7, vehicle control treated CIA animals had large inflammatory infiltrates in both ankles and knees, while treated animals had markedly reduced synovitis, consistent with the resolution of the inflammatory process in the treated groups as measured by both ^19^F MRI and clinical measurements.

## Discussion

^19^F MRI with a PFC contrast agent is emerging as an effective approach to evaluate the onset of inflammation in both acute and chronic diseases [28-34]. Our results extend these findings to the detection and evaluation of CIA, a model with a quantifiable clinical surrogate of disease severity, enabling a direct comparison of disease activity with the ^19^F signal. Numerous studies have co-located the perfluorocarbon reagent within macrophage at the site of inflammation, allowing ^19^F MRI to image a general characteristic of inflammation at the cell-function level [33,37]. The detection of the ^19^F signal in diseased animals in this and other studies [28-34] and the lack of ^19^F signal in naïve animals indicates the specificity of this imaging approach. However, it was not clear whether the intensity of the signal could be used as an independent measure of disease activity. This study is the first to extend previous findings of the presence of inflammation to show the potential of ^19^F MRI to reveal the severity of inflammation. More importantly, serial ^19^F MRI monitoring could effectively be used to evaluate the persistence of inflammatory responses, progression of disease, and longitudinal study of the response to therapy. Limitations of the present study include the lack of methods to detect PFC within individual phagocytes at the site of inflammation histologically, and the relative insensitivity of MRI to detect very low amounts of ^19^F which might be present at sites of minimal but potentially relevant inflammation, leading to a false negative. We have recently developed a dual mode fluorescent version of the PFC contrast agent which will facilitate the evaluation of specific cells containing the contrast agent in future studies. The data reported here indicate the utility of PFC contrast agent with ^19^F MRI for monitoring the course of disease to assess the efficacy of a therapeutic.

Early in the disease process, a marked difference between individual animals was found, both in disease severity as well as in the accumulation of contrast agent. A linear relationship was observed between the amount of contrast agent at the site of inflammation and a clinical measurement of the severity of the experimental disease. In two subjects a high accumulation of contrast agent appeared in the tail (Figure 3B), with a lower level of ^19^F signal relative to ankle swelling measurement. Arthritis in the CIA model is typically restricted to the fore and hind limbs without axial involvement [35], and no clinical signs of disease were noted in the tails of any subjects. We surmised that signal in the tail could be a consequence of failure to completely deliver the contrast agent into the bloodstream, and the resulting misadministration enabled the local accumulation of the PFC emulsion at the site of injection, effectively reducing the amount systemically available to label circulating phagocytes. These results indicate that while the intensity of the ^19^F signal in the lesioned paws correlated with disease severity, care in administration of the contrast agent is necessary for the most reliable readout.

In the serial imaging studies, a difference in the pattern of ^19^F accumulation over time was found between the vehicle control and prednisolone treated cohorts. In the control cohort, ^19^F signal in the diseased limbs continued to accumulate upon repeated administration, whereas in the treated cohort, the signal remained stable over time, even after repeat administration of contrast agent. Histological and caliper measurements of ankle swelling point to continued infiltration of macrophage in the vehicle control cohort, consistent with ^19^F measurements. Histological endpoints and caliper measurements show fewer inflammatory cells and less swelling in the treated group, and a stable ^19^F signal. While ^19^F MRI did accurately reflect the abatement of macrophage infiltration to the site of inflammation (i.e., no increases in ^19^F were observed in treated animals), the persistence of signal after the departure of disease points to the need for future study and characterization of tissue clearance mechanisms and timescales of the ^19^F reagent. While a simple linear correlation between ankle swelling and ^19^F signals was not observed in the context of repeated administration of the contrast agent at days 22 and day 29 (data not shown), the ^19^F results nonetheless reflected the clinical responses, in which increases in ^19^F reflected disease progression and the inhibition of further ^19^F accumulation in animals undergoing successful therapy with a measureable clinical response. This data points to the utility of ^19^F imaging as a surrogate biomarker for evaluating therapeutic efficacy in RA.

While the CIA model in rats is largely restricted to the fore and hind limbs, and can be clinically assessed by measuring changes in ankle size, not all inflammatory diseases provide for a facile, rapid measurement of a response to a therapeutic drug [41]. Arthritis which affects the axial skeleton, such as ankylosing spondylitis or spondyloarthropathy, does not present simple external measurements for disease severity in preclinical models [42] and MRI is a standard clinical practice in the diagnosis of the disease [43]. In this case, the ^19^F MRI method of precisely measuring site-specific inflammation in vivo could enable an opportunity to facilitate study and treatment of disease, aiding the clinical development of therapeutics for ankylosing spondylitis and other inflammatory conditions [29,33,34].

As a preclinical tool, ^19^F MRI may have advantages over histological evaluation of tissues, given that a single, live animal may be imaged in less than one hour. In contrast, histology requires biopsy or necropsy of the particular tissues of interest, followed by fixation, preparation of frozen tissue blocks or paraffin embedding, slicing and mounting tissue sections, then staining and cover-slipping slides before the tissue is evaluated microscopically. ^19^F is taken up by macrophages in situ, and the signal intensity at sites of inflammation is directly related to the degree of cellular macrophage infiltration [29,31,33,34], providing a rapid means of assessing inflammatory infiltration. Further, MR methods provide more comprehensive information of the extent and location of inflammation compared with selected representative tissue sections evaluated by histology for phagocytic cells, although it may not replace detailed evaluation of cell subsets or subcellular biomarkers. MRI also allows longitudinal studies in the same animal over time, without biopsy or other invasive procedures, such as synovial aspiration [44]. Ultimately, this may speed the screening of inflammatory drugs against disease, particularly for those diseases where an external measurement on a live animal is unavailable. In the absence of imaging equipment, excised tissues may also be evaluated by ^19^F NMR spectrometers [28] for a more high throughput approach to quantitatively evaluate inflammatory lesions, with tissue potentially amenable to histology following NMR analysis.

While the goal of this study was to evaluate the imaging potential, there were several incidental findings. The detection of high amounts of ^19^F in the proximity of the injection site suggests that tail vein injection was less successful then one might have predicted, and that inclusion of the contrast agent could enable one to quantify misadministration. It was also noted that administration of multiple large doses of the PFC contrast agent occurred in the absence of anaphylaxis or adverse clinical effects. While more extensive preclinical toxicological safety testing are necessary prior to drawing conclusions, the results here contribute to the accumulating data regarding the safety of systemic PFC administration for imaging and other applications [45-47].

## Conclusions

Pairing a PFC contrast agent with ^19^F MRI *in vivo* enabled a highly specific indicator of disease activity that has a direct correlation with a clinical measurement (i.e., ankle swelling in the CIA model). Here, it is shown that ^19^F MRI with a PFC contrast agent is not only useful for identification of sites of inflammation in RA, but can serve as a quantitative indicator of disease activity, including detection of disease progression, or remission in response to a therapeutic when applied to longitudinal in vivo imaging studies. The ability to unambiguously discern the infiltrating inflammatory cells from other anatomical features is highly desirable for the ability to quantify and sensitively detect disease progression. As the ^19^F signal does not alter the ability to acquire typical anatomical ^1^H images, imaging of both inflammation and the unadulterated anatomical features are possible with this approach. This approach may facilitate the drug development process in evaluating the efficacy of novel therapeutic regimens for RA and other inflammatory-based diseases, and eventually enable image-guided interventions or inform the therapeutic decision process in the clinic.

## Abbreviations

MRI: Magnetic resonance imaging; PFC: Perfluorocarbon; CIA: Collagen induced arthritis; RA: Rheumatoid arthritis; ROI: Region of interest; ^19^F: Fluorine-19; ^1^H: Hydrogen-1; AUC: area under the curve.

## Competing interests

The authors, except ETA, are employees of Celsense, Inc. and receive salary and stock options. ETA serves as a paid consultant to Celsense and is a stock holder.

## Authors’ contributions

AB contributed to the design of the studies, production of the contrast agent, and contributed to analysis of the MRI data, including rendering 3D images, and quantification. AB was involved in the interpretation of data and revision of manuscript. BMH contributed to the design and analysis of the studies, assessment of the contrast agent and also was involved in drafting and revising the manuscript. ETA assisted with design and aided in preparation of the manuscript. AKW and CFO were responsible for the conception and planning of the experiments, and CFO helped to revise the manuscript. AKW directed the study, designed the experiments, contributed to the analysis and interpretation of the data, and drafted the manuscript. All authors have read and approved the final version of this manuscript.

## Supplementary Material

Additional file 1 **Table S1.** Longitudinal analysis of ^19^F signal in CIA rats. Click here for file
