# Supplementary material for: Visualizing arthritic inflammation and therapeutic response by fluorine-19 magnetic resonance imaging (19F MRI)
Source: J Inflamm (Lond). 2012 Jun 21;9:24. doi: 10.1186/1476-9255-9-24 (PMC3506445; doi:10.1186/1476-9255-9-24)
Supplement: Additional file 1 — Table S1. Longitudinal analysis of 19F signal in CIA rats. [file 1476-9255-9-24-S1.pdf]

Table 1. Longitudinal analysis of ^{19}F signal in CIA rats

Cohort	Day	Range (^{19}F x10^{19})			Average ± SD (^{19}F x10^{19})	*P* value*	
	15	0.14	to	11.2	5.17 ± 4.55	5.43x10^{-6} (15 vs 22)	
Vehicle	*22*	6.59	to	19.2	11.9 ± 4.71		1.93x10^{-6} (15 vs 29)
	29	12.8	to	21.9	17.3 ± 3.82	3.95x10^{-4} (22 vs 29)	
	15	0.17	to	12.1	4.57 ± 4.86	NS (15 vs 22)	
Prednisolone	*22*	0.70	to	13.1	4.85 ± 4.63		NS (15 vs 29)
	29	0.91	to	13.8	5.92 ± 5.56	NS (22 vs 29)	

*After testing for normal distribution, two-way ANOVA was used to compare statistical significance among groups ($P < 0.05$ considered significant). Tukey's two-tailed paired T-test was then used to compare the average signal between time points for each treatment group (critical *P* value < 0.0083). SD = standard deviation, NS = not significant .
